# Timelines of past events: Reconstructive retrieval of temporal patterns

**DOI:** 10.2478/v10053-008-0101-5

**Published:** 2011-12-08

**Authors:** Maria G. Carell

**Affiliations:** Department of Psychology, Umeå University, Sweden

**Keywords:** temporal processing, time estimation, event representation, timelines

## Abstract

Most naturalistic events are temporally and structurally complex in that they comprise a number of elements and that each element may have different onset and offset times within the event. This study examined temporal information processing of complex patterns of partially overlapping stimulus events by using 2 tasks of temporal processing. Specifically, participants observed a pantomime in which 5 actors appeared on the scene for different periods of time. At test, they estimated the duration each actor was present or reconstructed the temporal pattern of the pantomime by drawing a timeline for each actor. Participants made large errors in the time estimation task, but they provided relatively accurate responses by using the timeline as a retrieval support. These findings suggest that temporal processing of complex asynchronous events is a challenging cognitive task, but that reliance on visuo-spatial retrieval support, possibly in combination with other temporal heuristics, may produce functional approximations of complex temporal patterns.

## Timelines of Past Events: Reconstructive Retrieval of Temporal Patterns

Most people have a variety of tasks to complete during an ordinary day. Typically, these tasks are not serial in that they follow a sequential timeline. Instead, many everyday activities are partially overlapping with different onset and offset times, and their completion also requires monitoring, rescheduling, and updating. For example, one needs to remember to take medication before breakfast while preparing coffee and boiling milk, and later having a lunch with a colleague who reminds one that the meeting at 2 p.m. was postponed 2 hr. Most of these and other daily activities require some form of temporal orientation as they should be completed within a limited time window while completing other activities.

Somewhat paradoxically, the empirical study of psychological time is well over a century old (James, 1950/1890; Kant, 1781/1965) but its psychophysically-oriented paradigms of interval timing do not capture these complexities. One limitation of past timing research is that the level of temporal complexity is very low. Typically, participants observe a discrete event for a few seconds and they are instructed to make a judgment of its duration (for overviews, see Block & Zakay, 1996, 1997; Zakay & Block, 2004). In most cases, the observed effects are consistent with the existing theories of interval timing such as the scalar expectancy theory (e.g., Church, 2003) and the attentional-gating model of prospective timing (Block & Zakay, 1996; Zakay & Block, 2004). Yet, most psychophysical tasks of interval timing are not easily applied to more complex goal-directed activities in everyday situations.

Real-world activities constitute a continuous stream of information that can be segmented into events and sub-events at multiple timescales (Zacks, Speer, Swallow, Braver, & Reynolds, 2007; Zacks & Tversky, 2001; see also Zalla, Pradat-Diehl, & Sirigu, 2003). Most naturalistic events are temporally and structurally complex in that they comprise a number of elements that may have different durations within the event.

Consider, for example, a theatre play in which the actors constitute the elements of the event (along with props). A theatre play reflects different temporal levels, including the real time of the performance, which commences at a specific hour and ends a couple of hours later. Furthermore, most events are composed of sub-elements, with individual temporal characteristics. In a play, the actors may enter and leave the scene simultaneously or separately and they may appear for different periods of time. For example, the actor A enters the scene first, after which the actors B and C enter, D appears then briefly, and A and C leave the scene together, followed by E, after which B leaves the scene, etc. In other words, the play (or any other dynamic event comprising multiple elements with different onset and offset times) does not only reflect its total duration, but the actions of individual elements constitute a temporal pattern of event information.

Although most real-world events can be considered complex patterns of sub-events with multiple timescales, research on multiple duration judgments is virtually nonexistent. To the best of my knowledge, only two studies have examined cognitive timing in the context of multiple duration judgments (Brown & West, 1990; Vanneste & Pouthas, 1999). Both studies tested the hypothesis that prospective timing requires attentional resources, and that task-irrelevant temporal information impairs prospective duration judgments. Thus, the primary focus of these studies was on the effects of concurrent temporal load on single-item duration judgments rather than on patterns of temporal information.

Specifically, Brown and West’s (1990) participants monitored the duration of one to four target stimuli with different onset and offset times. At test, they reproduced one of the durations. As can be expected, the main finding of their study was that the magnitude of prospective timing error increased as the number of stimuli increased from one to four targets. Vanneste and Pouthas (1999) extended these findings by showing that this effect was accentuated in older adults. Both studies were interpreted in terms of the attentional-gating model of prospective timing, supporting the view that cognitive timing requires attention. 

Consistent with the attentional-gating model, one implication of these findings is that people have great difficulties in keeping track of multiple temporal elements with different onset and offset times. On the other hand, real-world events do comprise multiple asynchronous elements, and most people seem to demonstrate quite good sense of time when completing everyday activities. A reasonable assumption is that subjective experience of time in these activities is based on a variety of temporal cues and multiple levels of temporal information (Block, 1989; Block & Zakay, 1996, 1997). Instead of internal timekeepers, a more economic strategy might be to rely on different forms of temporal heuristics, including lower-level temporal information (e.g., order information; see also Mäntylä, Carelli, & Forman, 2007), task-relevant knowledge structures (e.g., scripts and story schemata; Nelson, 1996), and spatial support systems (Casasanto & Boroditsky, 2008; Vallesi, Binns, & Shallice, 2008). A reasonable assumption would be that these temporal aids and heuristics are used to reconstruct and constrain the temporal pattern of the observed event (see also Block, 1989; Friedman, 1993, 2004; Friedman, Gardner, & Zubin, 1995).

For example, a witness may (incidentally) observe an event in which a series of persons and activities appear for different periods of time. The witness observes certain temporal and nontemporal attributes (e.g., who came first, who was seen alone or together with someone else, etc.), but would not be able to provide explicit duration judgments (e.g., “how long have you been seeing the person with the bag?”). However, an incorrect duration judgment might underestimate the witness´ actual competence. The witness might be able to provide a reasonable duration estimation by first reconstructing the temporal pattern of the observed event, possibly by relying on spatial recoding, and then using that construction or a “timeline,” as a form of retrieval guide (Mäntylä, 2010; see also Forman, Mäntylä, & Carelli, 2011; Mäntylä, Carelli, & Forman, 2007).

Following this line of reasoning, the aim of this study was to examine temporal processing of complex, partially overlapping event information. Instead of considering multiple durations as temporal distractors (Brown & West, 1990; Vanneste & Pouthas, 1999), our main interest was on temporal patterns per se, the primary focus being on mechanisms underlying multiple duration judgments. Specifically, we examined temporal processing of complex (asynchronous) event attributes by contrasting a traditional, psychophysically-oriented times estimation task with a more reconstructive cognitive timing task. We reasoned that retrieval of complex temporal events might be mediated by spatial representations. Specifically, participants first observed a pantomime, in which five actors entered and left the scene at different times. At test, they first completed two tasks of temporal processing. The first task was a traditional time estimation task, in which participants estimated the appearance time of each actor. In the second, timeline task, they reproduced the temporal pattern of the pantomime by drawing a timeline for each actor.

As noted earlier, past studies and the dominating models of interval timing suggest that representation of multiple, asynchronous durations is a very challenging (or even impossible) task in some conditions. Thus, the time estimation task was included here as a reference measure, while our primary goal was to examine whether temporal processing of complex events could be solved by relying on timeline-like retrieval support. Specifically, our primary hypothesis was that the time estimation task would produce substantial timing errors because asynchronous stimulus durations are not easily encoded and not easily handled by the models of interval timing (e.g., Block & Zakay, 1996; Church, 2003; Zakay & Block, 2004). However, and following the reasoning outlined earlier, we expected that the conventional measures of interval timing would underestimate participants´ temporal event knowledge and that the timeline task would serve as an efficient retrieval support for producing reasonable approximations of complex temporal patterns.

## Method

### Participants

Sixty Umeĺ University undergraduates (31 females and 29 males) participated in the experiment. They were between 20 to 28 years of age (*M*_age_ = 24.3, *SD* = 3.05), and none of them had prior experience of similar experiments.

### Stimulus and materials

**Figure 1.**
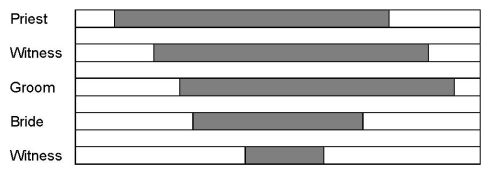
A timeline of the pantomime.

The stimulus event comprised a pantomime describing a wedding ceremony in which five actors (a priest, a bride, a groom, and two witnesses) were dressed in different clothes and colors (black, white, blue, green, and yellow t-shirts, respectively). The pantomime improvised the basic script of a wedding ceremony in that the priest first entered the scene where she performed a series of activities (arranging a few things in the room, opening a book, fixing her hair, etc.). Then the first witness appeared, followed by the bride and the groom. Finally, the second witness arrived and started a lively “conversation” with the other witness. Then one of the witnesses suddenly left the scene followed by the bride. The groom was comforted by the priest, who then left the scene. Finally, the groom left the scene, and the experimenter appeared for the test instructions. The duration of the whole event was 3 min15 s. The temporal structure of the event is illustrated in Figure 1.

### Procedure

The experimenter informed participants, who were tested simultaneously in a large room, that they would be shown a short pantomime in which a group of actors would perform a series of activities. Participants were informed that the pantomime depicted a “wedding ceremony,” and that each actor would be wearing different clothes and other accessories and that they would appear for different periods of time. Participants were instructed to pay attention to the whole event for a later memory test. The experimenter also informed that the play would start when the experimenter left the scene and stop when she returned to the scene. The whole event was video recorded, and the time codes of the video were used as a criterion for calculating timing errors.

After observing the play, participants completed the two timing tasks. A separate pilot study had indicated that time estimation performance improved when this task was completed after the timeline task, but not vice versa. To reduce these carry over effects, the two tasks were completed in the same fixed order, starting with the time estimation task. In this task, participants were instructed to indicate for how long time each actor appeared on the scene. Participants responded by writing a numeric value (in seconds) on a response sheet. In the timeline task, participants were instructed to draw a timeline corresponding to the start and stop times of each actor. They completed the task using a response sheet with a vertical “start line” and five horizontal “tracks” for each actor. Another vertical “stop line” indicated the total duration of the event (without providing any numeric values of its duration). The experimenter explained that the length of the “track” represented the total duration of the event, and that participants should estimate when and how long each actor appeared on the scene by drawing separate timelines for them. Participants were free to order the colors (indicated on the response sheet) as they preferred, but most participants listed the actors in the order of appearance. The experimenter clarified the test instructions by illustrating the task on a whiteboard. None of the participants appeared to have difficulties in understanding the instructions. The maximum response time for each task was 5 min and the whole experiment took about 20 min to complete.

## Results

The timing data of both tasks were analyzed in terms of absolute and relative errors. The former measure, referred to as the *absolute timing error*, was obtained by calculating the absolute difference between the observed and expected (actual) durations for each stimulus actor. For example, if the expected time was 95 s and the observed time was 80 s, then the absolute error would have been 15 s. *Relative timing error* was based on a ratio between the expected and observed duration (e.g., 95/80 = 1.19). This measure provides a standard score across the different time intervals, with coefficients above 1.0 reflecting overproductions and coefficients below 1.0 reflecting underproductions (see also Brown, 1985; Carelli, Forman, & Mäntylä, 2008). The timeline data was obtained by first transforming each response time to time units (where 10 mm = 6 s), and then calculating absolute and relative timing errors as indicated above.

**Figure 2.**
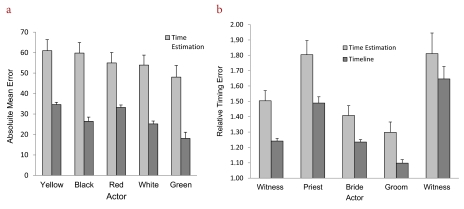
Absolute (a) and relative (b) timing error (in seconds) as a function of task and actor. Error bars refer to standard error of measurement.

Figure 2a shows the absolute timing data as a function of actor and timing task. As expected, these data suggest that participants were rather inaccurate when estimating the duration of each actor’s performance. The mean error of the time estimation task was large (*M* = 55.54) considering that the actual mean duration of the actors was 77 s (varying between 26 s and 103 s). By contrast, as shown in Figure 2a, the timeline task produced more accurate responses in that the absolute error rate (*M* = 27.50) was about 50% lower than that of the time estimation task. The relative timing data, shown in Figure 2b, suggested that both task conditions produced overestimations and that these errors varied across the five actors. Overall, the magnitude of relative timing error was greater in the time estimation task (*M* = 1.56, *SD* = 0.07) than in the timeline task (*M* = 1.34, *SD* = 0.02). Separate analyses of the timing data showed significant differences between the tasks for absolute errors, *F*(1, 59) = 39.27, *MSE* = 726.08, *p* < .01, and relative errors, *F*(1, 59) = 5.43, *MSE* = 0.11, *p* = .02. It should also be noted that the correlation analyses of the time estimation and timeline data indicated a nonsignificant association. Specifically, the (Pearson) correlation for the absolute errors was .02, and the corresponding value for the relative errors was .14 (*p* = .24).

## Discussion

The starting point of this study was the observation that past timing research is not easily applied to complex everyday tasks, which often involve multiple activities with different onset and offset times. In this study, we examined the hypothesis that temporal information processing of complex event information is based on reconstructive retrieval operations, rather than on timing of absolute durations. We hypothesized that, instead of relying on multiple mental clocks and costly computations of time setting and monitoring, a more flexible strategy might be to use a variety of temporal aids and heuristics in order to reconstruct and constrain the temporal pattern of the observed event. The timeline task was used as a procedure for examining these reconstructive processes in experimental settings.

We contrasted the timeline task with a more traditional time estimation task in a setting in which participants viewed a relatively complex pattern of partially overlapping stimulus durations. Participants of the present study were cognitively competent university students who were instructed to pay attention to the temporal pattern of the stimulus event information. Yet, they made very large errors in the time estimation task. By contrast, when using the timeline procedure, participants produced relatively accurate responses. Thus the findings of this study supported our hypothesis that participants made larger errors in the time estimation task than in the timeline task.

The two timing tasks showed large differences in accuracy, but it should be noted that they were based on the same encoding phase and study instructions (manipulated within subjects). In both tasks, the five target durations were reported simultaneously, could be related to each other, and modified during the course of retrieval. Furthermore, the two tasks involved virtually identical scoring procedures with comparable and multiple measures of timing error.

 Our findings are consistent with those of Brown and West (1990) and Vanneste and Pouthas (1999), and suggest that time estimation of multiple durations is a demanding task in that participants were not able to report temporal event information in terms of absolute durations. However, when using the timeline procedure they were quite good at reconstructing temporal patterns of relative positions and overlaps. 

A reasonable interpretation of these findings is that participants did not represent the start and stop times of each actor in terms of absolute intervals. Instead, the timeline data suggest that they reconstructed an approximation of these durations, possibly by constraining the relative position of each individual element by means of lower-level temporal (order) information in combination with semantic knowledge structures and spatial “scaffolding.” The correlation data are also consistent with this notion in that accurate performance in the time estimation task was not associated with good performance in the timeline task, and vice versa. Although these correlations should be interpreted cautiously they might indicate that the two timing tasks are mediated by different mechanisms (cf. Block & Zakay, 1996, 1997; Brown, 1985).

As noted earlier, it is reasonable to assume that processing of complex events is based on a variety of cues and temporal heuristics, including task-relevant knowledge structures, such as scripts and story schemata (e.g., Nelson, 1996). An interesting avenue for future research would be to examine the role of prior knowledge and expectations on representation of complex temporal patterns. A reasonable hypothesis here would be that schema consistency contributes to encoding of complex temporal patterns. Participants of this study were expecting a wedding ceremony and probably used that knowledge structure in order to encode the sequence of actors appearing in the improvised pantomime (see also Carelli & Mäntylä, 1997). One way of examining this hypothesis in more detail would be to manipulate schema consistency (i.e., participants’ expectations) or event structure (i.e., meaningfulness of the event).

 The present findings are consistent with the notion that processing of temporal information is, at least to some extent, mediated by spatial representations. Evidence from psychophysical experiments (Casasanto & Boroditsky, 2008; Vallesi, Binns, & Shallice, 2008) and psycholinguistic studies (e.g., Boroditsky, 2000; Núńez & Sweetser, 2006; Tversky, Kugelmass, & Winter, 1991) suggests that people construct spatial representations online when processing temporal information and that this relationship is asymmetric (Boroditsky, 2000; Casasanto & Boroditsky, 2008). For example, Casasanto and Boroditsky (2008) used a temporal reproduction task, in which the duration of a line (or a dot) was varied continuously and orthogonally with its left-to-right spatial displacement. Participants had to reproduce either temporal duration or spatial displacement. Casasanto and Boroditsky (2008) found that the irrelevant spatial displacement influenced the reproduction of temporal duration, but not vice versa, suggesting that mental representations of duration and spatial displacement are asymmetrically dependent on one another. 

The timeline task, which can be considered as a form of spatial visualization aid, does not require a direct translation of duration experience to conventional units of time, such as seconds and minutes in the time estimation task. Instead, subjective experience of stimulus durations is represented in terms of relative positions, and the resulting pattern of timelines can be used to access more absolute duration estimates. This characteristic of the timeline task might make it particularly suitable for examining temporal information processing in certain populations (see also Friedman & Kemp, 1998; Piaget, 1927/1969).

Taken together, the present study suggests that timing of multiple event attributes is a challenging task when considered in terms of pacemaker models of interval timing. However, a more contextual and memory-based approach relying on spatial retrieval support, possibly in combination with other heuristics, may produce representations that are reasonable approximations of complex temporal patterns, and thereby provide a functional basis for sense of time in everyday activities.

## Author Note

This research was supported by a grant from the Swedish Research Council. Parts of this paper were presented at the Annual Meeting of the Psychonomic Society, Long Beach, CA (November, 2008).
